# Augmented annotation and orthologue analysis for *Oryctolagus cuniculus*: Better Bunny

**DOI:** 10.1186/1471-2105-13-84

**Published:** 2012-05-08

**Authors:** Douglas B Craig, Alan A Dombkowski

**Affiliations:** 1 Department of Pediatrics, Wayne State University School of Medicine, Detroit, MI, USA; 2 Department of Anesthesiology and Critical Care Medicine, Johns Hopkins University, Baltimore, MD, USA

**Keywords:** Rabbit, Genome, Annotation, Orthologue, Microarray, Functional analysis

## Abstract

**Background:**

The rabbit is an important model organism used in a wide range of biomedical research. However, the rabbit genome is still sparsely annotated, thus prohibiting extensive functional analysis of gene sets derived from whole-genome experiments. We developed a web-based application that provides augmented annotation and orthologue analysis for rabbit genes. Importantly, the application allows comprehensive functional analysis through the use of orthologous relationships.

**Results:**

Using data extracted from several public bioinformatics repositories we created Better Bunny, a database and query tool that extensively augments the available functional annotation for rabbit genes. Using the complete set of target genes from a commercial rabbit gene expression microarray as our benchmark, we are able to obtain functional information for 88 % of the genes on the microarray. Previously, functional information was available for fewer than 10 % of the rabbit genes.

**Conclusions:**

We have developed a freely available, web-accessible bioinformatics tool that enables investigators to quickly and easily perform extensive functional analysis of rabbit genes (http://cptweb.cpt.wayne.edu). The software application fills a critical void for a wide range of biomedical research that relies on the rabbit model and requires characterization of biological function for large sets of genes.

## Background

The rabbit (*Oryctolagus cuniculus*) is an important model organism used extensively in biomedical research. Offering advantages over other organisms for a variety of physiological systems, the rabbit has contributed to studies in a wide range of disciplines, including embryology, ophthalmology, toxicology, pulmonary and cardiovascular research, and neurology [1-4]. For example, the rabbit has proven to be invaluable in neurodevelopmental research, largely due to a temporal pattern of oligodendrocyte maturation and myelination that closely parallels that of humans. In rabbit brain development structural and functional changes occur most rapidly during the perinatal period, generally starting several days before birth and continuing in the postnatal period [5]. This pattern is very similar to what occurs in humans, where immature oligodendrocytes increase rapidly in number in the third trimester followed by myelination that initiates around the time of birth, with maximum increases during the first year of life [6]. Thus, structural development of the rabbit brain is very similar to that of humans, but within a more compressed time-frame. In contrast, rodents are postnatal brain developers, while sheep, pigs and monkeys are prenatal brain developers. Consequently, the rabbit offers a preferred means to investigate the effects of perinatal events and environmental exposures on brain development. This model has been used extensively in one of our own laboratories to study the relationship between maternal infection and neonatal brain injury [7,8], and by other investigators to study the effects of various prenatal and perinatal insults to the brain [9].

Despite the demonstrated importance of rabbits in biomedical research, the rabbit genome is still poorly annotated. This presents a significant challenge where whole-genome technologies are used to characterize molecular and cellular events. Using high-throughput genomic methods such as microarrays, investigators often obtain lengthy lists of differentially expressed genes, and a commonly employed analysis paradigm is to perform functional annotation analysis to identify enriched ontologies, pathways, motifs, and other molecular details associated with a set of genes. A variety of software is available for functional analysis of common model organisms; however, this approach is currently impeded when using rabbit data due to sparse annotation that is scattered among various bioinformatics resources. Only 1076 rabbit genes are annotated with biological process information per the current database release of the Gene Ontology Consortium (GO) [10]. This compares to more than 27,000 genes with an annotated biological process in human, and approximately 16,000 and 24,000 genes in rat and mouse respectively. Additionally, the rabbit is not well represented in other informatics resources. For example, rabbit is not included in the Kyoto Encyclopedia of Genes and Genomes (KEGG) which is widely used for biological pathway analysis [11]. These limitations are further exacerbated by the absence of rabbit in Homologene [12], an NCBI resource that provides orthologous relationships among model organisms. Such relationships can be effectively used to infer functional details for genes having little annotation, where an orthologue is better characterized in another organism. In the case of rabbit, one could utilize the well-annotated and highly homologous genes from human, mouse, and rat for inferred functional annotation. However, at this time investigators who depend on the rabbit model for biomedical studies are confronted by the obstacle of a time-consuming informatics effort to assemble pertinent annotation and orthologue data, consequently they risk overlooking potentially important findings in their experiments.

The need for a freely available tool to perform functional analysis of rabbit genes is highlighted by a recent report that used a rabbit model to study the global gene expression response to *Mycobacterium tuberculosis* infection and investigate the influence of immune system modulation on treatment efficacy [4]. The authors used Agilent rabbit gene expression microarrays to measure genome-wide expression changes in lung tissues. After obtaining lists of differentially expressed genes through statistical analysis of the microarray data, the authors sought to perform functional classification of the identified genes. However, they noted that functional annotation of most rabbit genes is currently unavailable. So they performed extensive computational work to extract orthologous relationships from a commercial bioinformatics database, which were then used to infer the function of rabbit genes based on annotation of their human, mouse, and rat counterparts. While the authors of the study were successful using this approach, many research groups do not have access to such proprietary data and/or the computational expertise to derive a functional annotation database. To enable investigators to perform functional analysis of rabbit genes, we have created a web-accessible functional annotation tool using data derived from public repositories. The application is easy to use and freely available.

## Implementation

To address the paucity and dispersed state of available rabbit annotations, with the ultimate goal of functional analysis, we have created a web-based application for augmented annotation and analysis of rabbit genes: “Better Bunny.” Access to the web server is freely available at http://cptweb.cpt.wayne.edu. The server provides annotation and orthologous relationships assembled from public bioinformatics resources, including NCBI [13] and Ensembl [14] databases. Importantly, comprehensive pathway, ontology, and functional analysis can be instantly performed on rabbit gene lists using orthologous relationships provided through Better Bunny.

**Figure 1:**
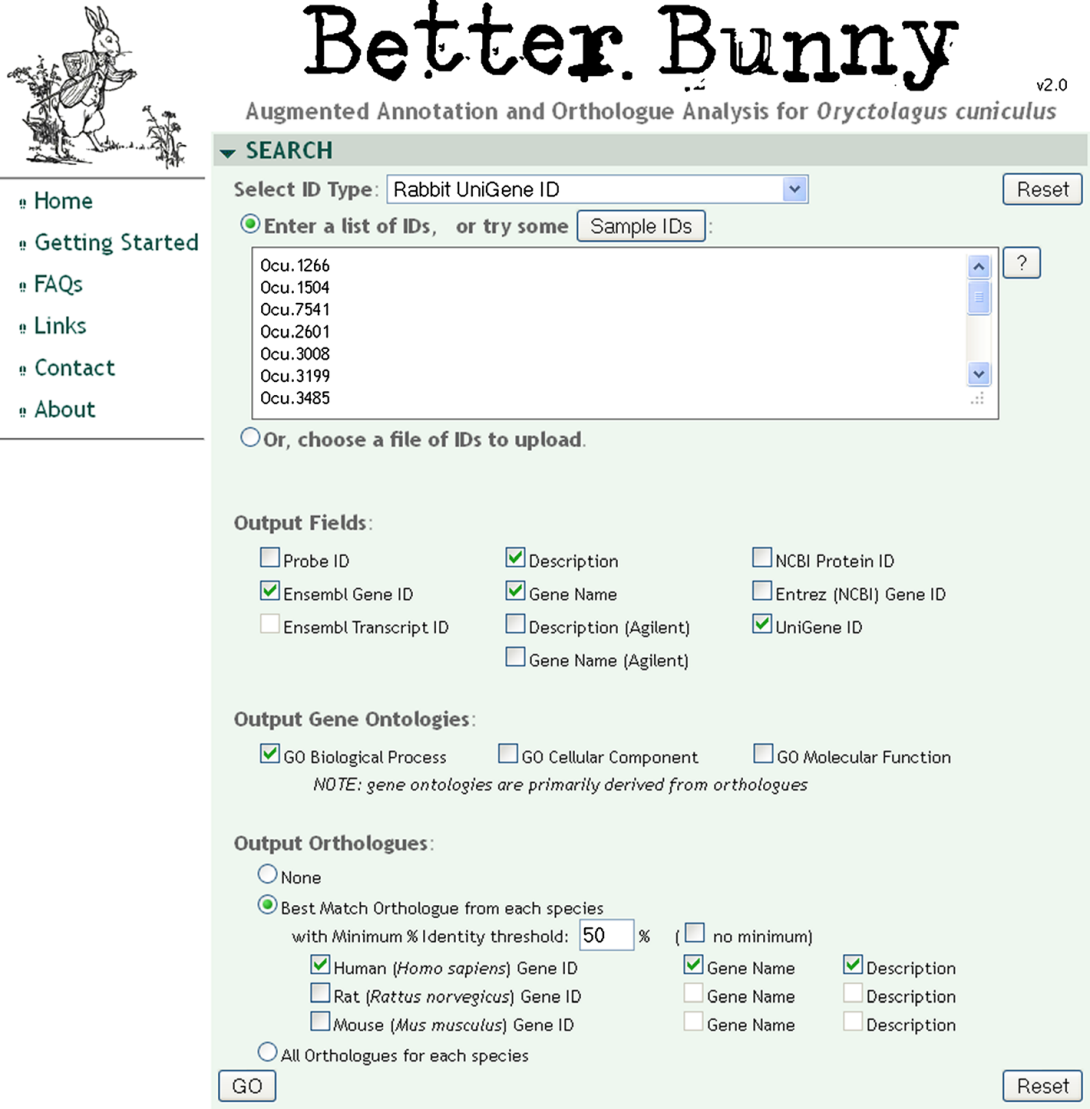
**The Better Bunny query page**. Better Bunny provides an easy to use interface that accepts several rabbit gene identifiers as input. The user can select from a range of available output, including ontology classification and putative orthologues in human, mouse, and rat.

**Figure 2:**
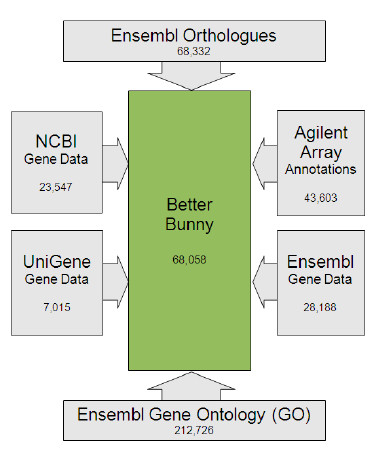
**Better Bunny architecture**. Better Bunny integrates all available rabbit data from Ensembl, NCBI, and Agilent (gene expression microarray) for rabbit gene annotation and augmentation. Data sources are shown along with the number of records drawn from each source.

Better Bunny is easy to use, allowing the user to submit a list of rabbit gene identifiers and to select from a range of available annotation options for output. The Better Bunny query page is shown in Figure 1. A helpful guide to using Better Bunny is available under the “Getting Started” link, and additional information is found under the “FAQ” link. The input gene set might be, for example, those identified as differentially expressed from a microarray experiment and could include thousands of genes. Several types of gene identifiers are allowed as input: NCBI Unigene, NCBI Entrez, NCBI protein, Ensembl gene IDs, and Agilent rabbit microarray probe identifiers. A wide range of data from NCBI, Ensembl, and Agilent have been integrated and cross-referenced within Better Bunny to provide a comprehensive resource, as shown in Figure 2. We have included gene annotation, ontology, and orthologue data for all rabbit entries in Ensembl. Likewise, we have included Entrez gene, Unigene, and protein data for all rabbit entries within the NCBI resources. Better Bunny also includes all annotation available from Agilent Technologies for target genes of the only commercial gene expression microarray that is currently available. Cross-referenced within Better Bunny, the aggregate of these data are provided in response to user queries. Check boxes enable the selection of the desired output, including gene descriptions, ontologies, orthologues, and accession numbers. Original Agilent array descriptions and gene names are also available for output. Most output identifier fields include hyperlinks to their respective source database. Variant annotation values, for example gene names, can always be found in the source data repository by following these hyperlinks. Search results can be exported as a comma delimited file for use in any spreadsheet program.

**Figure 3:**
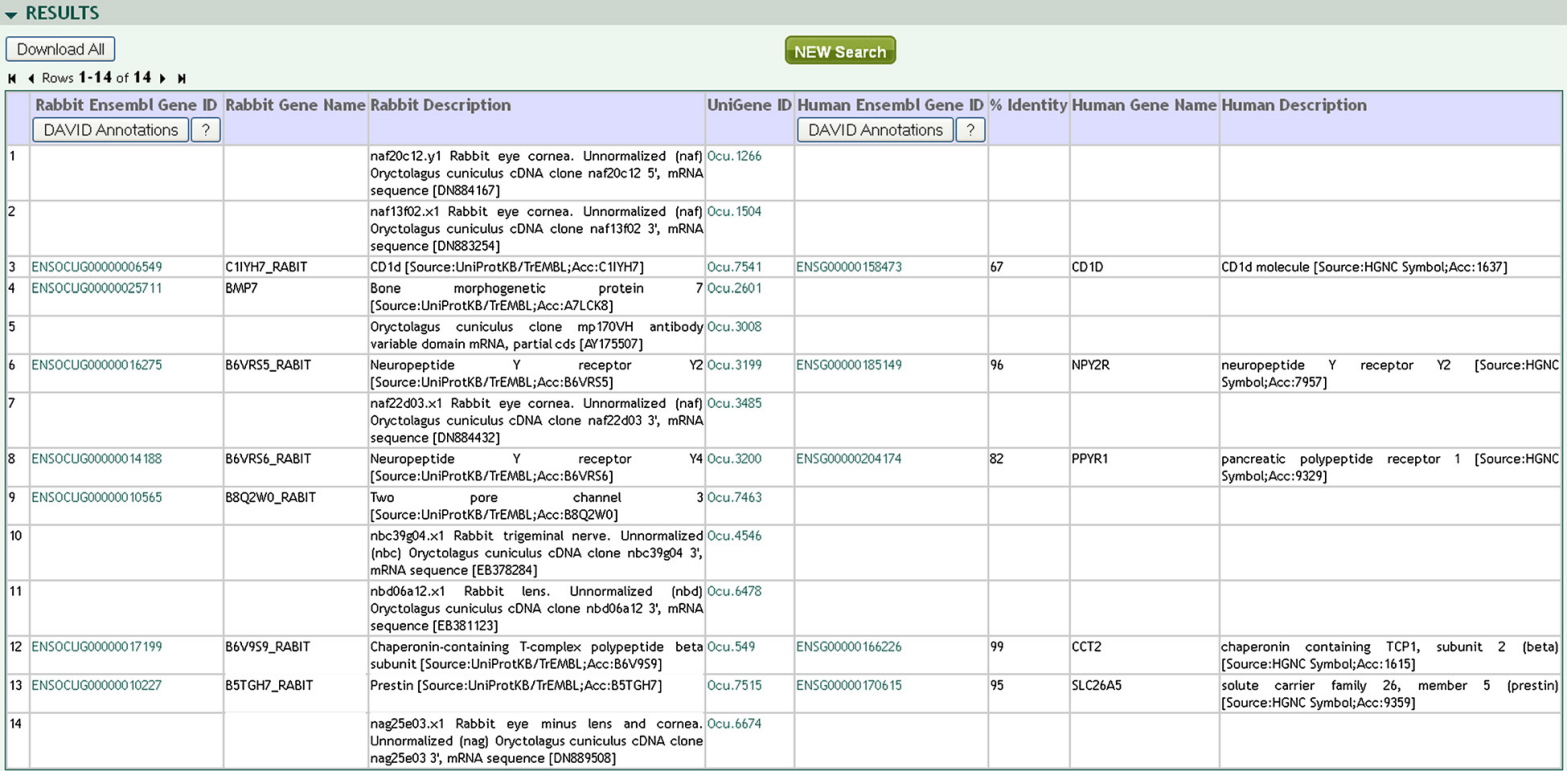
**Example of Better Bunny output.** Each row represents an input query gene, and each column provides a selected annotation element. Hyperlinks are available to Ensembl resources. Gene identifiers from selected orthologue species are provided, and the output gene sets can be seamlessly submitted to DAVID with one mouse click for extensive functional analysis.

An important feature of Better Bunny is the ability to identify orthologues for queried rabbit genes. Very little functional annotation is available in public databases for rabbit genes. Therefore, the use of orthologues allows investigators to perform functional annotation that may otherwise be unavailable. Orthologues can be selected from human, mouse, and rat. The orthologous relationships in Better Bunny are derived from Ensembl and are based on protein sequence similarity. Ensembl employs an automated orthologue identification pipeline that performs pairwise alignments (BLASTP) between all pairs of proteins for all available species [15]. The alignment is performed using the longest protein translation for each gene, and the percent identity displayed in Better Bunny is the value relative to the target (non-rabbit) species. Functional annotation transfer based on sequence similarity is widely used to establish putative function for poorly characterized proteins. While high sequence similarity is not a guarantee of similar function, studies have demonstrated that a threshold of 50-60 % identity ensures a high probability of similar function at the enzyme and gene ontology levels [16]. Better Bunny allows users to specify a minimum required percent identity for the selection of orthologues. An individual rabbit gene may have multiple orthologues in another genome that meet the minimum percent identity requirement. The user can specify whether the output is to include all orthologues meeting the minimum identity requirement, or only the single orthologue having the highest percent identity for each gene. An example of Better Bunny output is shown in Figure 3. Each row represents a gene from the input set, and each column provides a selected annotation element. Each selected orthologue species will have a corresponding set of annotation columns.

One of the most comprehensive and widely used applications for functional annotation analysis is the Database for Annotation, Visualization and Integrated Discovery (DAVID) [17,18]. This well-maintained resource provides extensive functional analysis of input gene sets, including enrichment of pathways and ontologies, annotation clustering, and identification of protein interactions and domains. We have seamlessly integrated DAVID analysis into the Better Bunny application. The output from Better Bunny includes a “David Annotations” link at the top of the Ensembl Gene ID column for each species selected. This link automatically submits the gene list from the selected species to DAVID and opens a new browser window with the results. Adjacent to the “David Annotations” link is a “?” that provides information on using DAVID for functional annotation. Readers are encouraged to explore the ample help available at the DAVID web site for additional information on this valuable resource. While very little functional information is typically obtained when rabbit gene identifiers are submitted to DAVID, using orthologues via Better Bunny quickly provides a rich source of inferred functional information for most rabbit genes in a list.

## Results and discussion

To assess the value of Better Bunny in the context of a whole-genome experiment, we examined the annotations associated with the entire set of probes on the Agilent rabbit gene expression oligonucleotide microarray. There are 43,603 probes on the microarray, with many genes targeted by redundant probes. The following discussion reflects annotation of the unique set of target genes. Of the 12,118 unique genes represented on the microarray (based on Ensembl gene ID), only 916 genes have a gene name per the vendor’s annotation, 2324 have a Unigene identifier, and no gene ontology information is provided. The remaining genes include many uncharacterized clones. Using the augmented annotation available through Better Bunny, more than 10,000 genes are provided a name, largely through Ensembl resources, and 9470 genes have a molecular function assigned from the Gene Ontology Consortium, most established by Better Bunny through orthologous relationships.

Of great utility, Better Bunny identifies 11,026 putative human orthologues with at least 50 % identity to the corresponding rabbit sequence, and a similar number are available for mouse and rat. This enables instant and extensive functional annotation for most genes on the microarray via the integrated DAVID analysis available in Better Bunny. To demonstrate the functional information that is gained through Better Bunny, we submitted the probe list for all genes on the Agilent microarray to Better Bunny and specified the output to include human orthologues having a minimum of 50 % identity. The set of 11,026 orthologous human genes was then submitted to DAVID using the integrated link available in Better Bunny. Using default settings in DAVID, functional annotation was obtained for 10,731 genes, representing a wide range of functional information. For example, SwissProt (UniProt) comments were obtained for 10,221 genes; Gene Ontology Biological Process annotation was obtained for 8285 genes; 3184 genes were associated with KEGG pathways; and Interpro protein classification information was obtained for 9697 genes. This compares to only one rabbit gene with functional annotation when rabbit gene identifiers are submitted to DAVID. The dramatic difference in results obtained for the two species reflects the scarcity of rabbit gene annotation available in the underlying databases, as exemplified by the GO biological process annotation for rabbit which currently covers only approximately 1,000 genes, and the results demonstrate the benefit of using orthologous relationships for functional annotation.

## Conclusions

In summary, we have developed a freely available and web-accessible bioinformatics tool that enables investigators to quickly and easily perform extensive functional characterization of lengthy lists of rabbit genes. The software application fills a critical void for investigators who employ the rabbit model in their research and who wish to characterize the biological function and cellular role associated with sets of genes. Using the complete set of target genes from a commercial rabbit microarray, we were able to obtain functional information for 88 % of the genes, whereas functional information was previously available for fewer than 10 % of the rabbit genes on the microarray. The augmented gene annotation, orthologue identification, and integrated functional analysis available through Better Bunny are expected to greatly enhance the knowledge gained from a wide variety of biomedical research projects using the rabbit model.

## Availability and requirements

**Project name:** Better Bunny

**Project home page:**http://cptweb.cpt.wayne.edu

**Operating system(s):** Platform independent

**Programming language:** PHP / Python / MySQL

**Other requirements:** none

**License:** none

**Any restrictions to use by non-academics:** none

## Competing interests

The authors declare that they have no competing interests.

## Authors' contributions

DC, SK, and AD contributed to the software design and testing. DC implemented the software. All authors drafted, edited, and approved the final manuscript.
